# 1-MCP maintains postharvest quality in winter jujube during low-temperature storage by regulating energy and sugar metabolism and enhancing antioxidant capacity

**DOI:** 10.3389/fpls.2025.1702030

**Published:** 2025-10-24

**Authors:** Wen Tang, Biao Xiang, Yang Cao, Cuiyun Wu, Chuanjiang Zhang, Minjuan Lin, Peihua Du

**Affiliations:** ^1^ College of Horticulture and Forestry, Tarim University/The National and Local Joint Engineering Laboratory of High Efficiency and Superior-Quality Cultivation and Fruit Deep Processing Technology of Characteristic Fruit Trees in Southern Xinjiang, Alar, China; ^2^ Southern Xinjiang Distinctive Foresty & Pomology Technology Innovation Center, Xinjiang Production and Construction Corps, Alar, China

**Keywords:** 1-methylcyclopropene, antioxidant capacity, energy metabolism, low-temperature storage, sugar metabolism, winter jujube

## Abstract

This study investigates the effects of 1-methylcyclopropene (1-MCP) treatment on postharvest storage of winter jujube. The results indicate that after 1-MCP treatment, the pyruvate (PA) content in winter jujube decreased by 20% at 30 days compared to the control. The energy charge (EC), ATP, and ADP levels increased by 7%, 17%, and 27%, respectively. Activities of key enzymes involved in energy metabolism, including succinate dehydrogenase (SDH), cytochrome c oxidase (COX), H^+^-ATPase, and Ca^2+^-ATPase were elevated. Furthermore, the activities of acid invertase (AI) and neutral invertase (NI) were 27% and 26% lower, respectively, than those in the control. Sucrose synthase (SS) activity increased by 52%, while the activities of hexokinase (HK) and phosphofructokinase (PFK) decreased by 19% each. Activities of key antioxidant enzymes-superoxide dismutase (SOD), peroxidase (POD), catalase (CAT), and glutathione reductase (GR)-were significantly enhanced. Non-enzymatic antioxidants, including vitamin C (VC), flavonoids, total phenols, and reduced glutathione (GSH) contents, were effectively retained, and suppressing the accumulation of the hydrogen peroxide (H_2_O_2_) and malondialdehyde (MDA). These findings suggest that 1-MCP treatment preserves the postharvest quality of winter jujube by enhancing energy metabolism, delaying sugar metabolism, and improving antioxidant capacity.

## 1 Introduction

Winter jujube (*Ziziphus jujuba* Mill. cv. ‘Dongzao’) is a leading cultivar of fresh jujube in China. The fruit is rich in bioactive compounds, including polysaccharides, vitamin C (VC), flavonoids, and total phenols (Zhao et al., 2019b; Sang et al., 2022). These active ingredients enhance immune function and possess antitumor and liver-protective effects (Sun et al., 2022; Kou et al., 2018). However, fresh winter jujube has a short postharvest life due to its crunchy, thin, and juicy characteristics (Hei et al., 2025). Therefore, numerous approaches have been used to prolong the storage time of winter jujube. Reducing the storage temperature is the simplest and most widely applied method (Zhang et al., 2024b).

With the increasing demand for the quality of agricultural products, low-temperature treatment alone can no longer ensure adequate quality preservation. Research is increasingly focusing on the application of technologies such as light irradiation, exogenous hormones, and preservatives (Frederike et al., 2023; Wei et al., 2024; Zhang et al., 2025a). Ethylene plays a significant role in fruit senescence, leading to quality deterioration in quality and decay, thus becoming a critical factor influencing fruit quality and shelf life (Li et al., 2022; Yang et al., 2022). 1-methylcyclopropene (1-MCP), which functions as an ethylene receptor inhibitor by irreversibly binding to ethylene receptors, has been extensively applied to delay fruit ripening processes (Brizzolara et al., 2020; Wang et al., 2025a). The primary effect of 1-MCP is to reduce ethylene production, thereby influencing various metabolic pathways and minimizing softening and decay (Zhu et al., 2020; Zhang et al., 2024a). In climacteric fruits such as ‘Dajie’ apricot and ‘SiLuHongYu’ fig, ethylene production increases rapidly after harvest. The application of 1-MCP effectively reduces ethylene synthesis and preserves fruit quality. Additionally, it is effective in maintaining the postharvest quality of non-climacteric fruits like ‘Kinnow’ mandarin and ‘Allen Eureka’ lemon (Liang et al., 2023; Wang et al., 2023; Baswal et al., 2020; Mitalo et al., 2020).

The imbalance of postharvest reactive oxygen species (ROS) metabolism is a critical factor contributing to senescence and quality deterioration (Zhou et al., 2025). Excessive accumulation of ROS damages the integrity of cell membranes, thereby adversely the postharvest storage quality (Hao et al., 2019). Both enzymatic and non-enzymatic antioxidant system work to reduce the excessive ROS after harvest (Liu et al., 2024). Applying an appropriate amount of 1-MCP to winter jujube can increase the activities of antioxidant enzymes such as superoxide dismutase (SOD), catalase (CAT), and peroxidase (POD), thereby reducing the excessive accumulation of free radicals and hydrogen peroxide (H_2_O_2_), maintaining the ROS balance, preserving overall fruit quality (Zhang et al., 2022a). Similar effects have been observed in ‘Ruiguang 7’ nectarine and ‘Nanguo’ pear, which also exhibited higher energy levels and ATPase activity (Zhang et al., 2020; Tao et al., 2019).

The postharvest energy status of cells serves as a critical regulatory factor in the processes of ripening and senescence. Higher ATP levels and energy charge (EC) contribute to mitigating cell membrane damage, thereby supporting cellular integrity (Cheng et al., 2015; Li et al., 2020). Simultaneously, the reduction of cellular energy levels helps alleviate cold stress and plays a pivotal role in regulating physiological metabolism (Li et al., 2024). The application of glycine betaine to winter jujube enhances the activities of succinate dehydrogenase (SDH), cytochrome c oxidase (COX), H^+^-ATPase, and Ca^2+^-ATPase, thereby maintaining energy levels and providing the energy required for antioxidation. Similarly, applying acidic electrolyzed water to ‘Lingwu long’ jujube produces comparable effects (Zhang et al., 2023; Jia et al., 2024). Chitosan coating applied to ‘Majiayou’ pummelo can reduce the energy consumption while maintaining the levels of metabolites in the tricarboxylic acid cycle (TCA) (Chen et al., 2021). The TCA, electron transport chain (ETC), glycolysis, and pentose phosphate pathway (PPP)-all key respiratory metabolic pathways in plants-are directly linked to energy metabolism (Tao et al., 2022). Meanwhile, the sugars accumulated during development, which serve as both an energy source and signaling molecules, remain stable or decrease during storage due to changes in the balance between synthesis and degradation, thereby regulating multiple physiological processes (Shuai et al., 2025). The soluble sugars in winter jujube primarily consist of glucose, fructose, and sucrose, and their levels continuously decrease during storage. Meanwhile, the excessive accumulation of anaerobic metabolites produced by anaerobic respiration in postharvest fruit is a typical characteristic of their senescence, which is closely related to glycolysis (Ji et al., 2023; Li et al., 2023a). However, there have been few reports on the study of energy metabolism and sugar metabolism in winter jujube treated with 1-MCP.

Based on the above literature, we hypothesize that 1-MCP maintains postharvest quality by enhancing energy production, suppressing sugar catabolism, and activating antioxidant defenses, thereby reducing oxidative damage and senescence. In this study, winter jujube was treated with 1-MCP and stored at 4°C to investigate postharvest preservation mechanisms. Dynamic changes in energy metabolism (ATP, ADP, key enzymes), sugar metabolism (soluble sugars, metabolic enzymes), and antioxidant capacity (antioxidant enzymes, ROS levels) were monitored. The aim was to clarify intrinsic regulatory relationships among these three interconnected metabolic pathways during low-temperature storage, providing theoretical support for optimizing winter jujube storage technology and extending shelf life.

## 2 Materials and methods

### 2.1 Materials and treatments

Winter jujube (*Ziziphus jujuba* Mill. cv. Dongzao) fruit with light-green and red colors were harvested in Alaer, Xinjiang, China (81°40’E, 40°38’N) in September. The harvested fruit were transported to the laboratory at 2 ± 2°C. After air-cooling at 0°C for 2 h, 30 kg winter jujube fruit of uniform maturity and size (free from mechanical damage) were selected. The selected fruit were randomly assigned to two groups, under storage at 4°C. Select the concentration of 1-MCP, based on previous research (Zhang et al., 2022a). One group was administered a fumigation treatment with 1 μL L^-1^ 1-MCP, while the other group as control. The duration of the treatment was 24 h. The pulp of fruit was collected every 5 days until 30 days, frozen in liquid nitrogen, and placed in a refrigerator at − 80°C for subsequent analyses. The relative humidity (RH) of storage environments was maintained at 90 ± 5%. Three biological replications were conducted independently for winter jujube fruit.

### 2.2 Determinations of decay and weight loss

Select 3 kg of winter jujube fruit, and then randomly assigned to three groups. After completing the following measurements, they were placed back in their original positions until the next measurement. The fruit surface with sunken, wrinkled areas, cracks and mold area were identified as decay fruit. The calculation formula of decay was as follows:


$$Decay=\frac{Total\;number\;of\;decay\;fruit}{Total\;number\;of\;fruit}$$

The weight loss was measured according to Jin et al., 2022. The calculation formula of weight loss was as follows:


$$Weight\;loss=\frac{0\;day\;weight-x\;days\;weight}{0\;day\;weight}$$

Where *x* means the value of 5, 10, 15, 20, 25, and 30.

### 2.3 Determinations of CO_2_ levels, ethylene levels, and firmness

The CO_2_ and ethylene levels measurements were conducted in airtight chambers of 2.5 L volume. Each chamber, equipped with a plastic diaphragm (with petroleum jelly applied at the interface), was filled with at about 300 g fruit (three biological replications), and maintained for 1 h. The measurements were performed using an Agilent 7890B gas chromatograph (Agilent Technologies Inc.). The column is HayeSep Q 80/100 (2 mm, 1 m×1.125 in, stainless steel). The findings were reported as nmol of CO_2_ and ethylene produced per kilogram per second, in accordance with the method of Spricigo et al., 2021.

Each measurement involves approximately 200 g of fruit (three biological replications). The fruit peel was removed out and measure firmness by TP-GY-4 (indenter Φ 3.5 mm).

### 2.4 Determinations of ATP, ADP, AMP, pyruvate (PA) contents, and EC

Homogenize 1 g of frozen fruit (from about 300 g fruit) with 5 mL of 0.6 mol L^-1^ perchloric acid, centrifuge (4°C, 10000 ×*g*) for 20 min, and collect the supernatant. Then, add 1 mol L^-1^NaOH to adjust pH to 6.8. Finally, pass through the 0.22 micrometer filter membrane. The measurements were performed using Shimadzu LC-20A. The column is AQ-C18 (5 μm, 4.6×250 mm, GL Sciences Ltd.), the detection wavelength set at 240 nm. The findings were reported as microgram of ATP, ADP, and AMP per gram, in accordance with the method of Zhao et al., 2019a.

The content of PA was determined according to the method proposed by Lv et al., 2021. Accurately weigh 1 g of the sample and mix it with 8% trichloroacetic acid, then centrifuge. To the supernatant, sequentially add 0.1% 2, 4-dinitrophenylhydrazine, 8% trichloroacetic acid, and 1.5 M sodium hydroxide. Measure the absorbance at a wavelength of 520 nm. The calculation formula of EC was as follows:


$$EC=\frac{ATP+0.5\times ADP}{ATP+ADP+AMP}$$

### 2.5 Determinations of SDH, COX, H^+^-ATPase, and Ca^2+^-ATPase activities

Activities of SDH and Ca^2+^-ATPase were determined by assay kit (item number: BC0955 and BC0965, Beijing Solarbio Science & Technology Co., Ltd.). Weigh precisely 0.2 g of the sample, grind it, and then follow the standard kit instructions. One unit of Ca^2+^-ATPase activity is defined as the release of 1 μmol of phosphorus per milligram of tissue protein per min.

Activities of COX and H^+^-ATPase were determined according to the method of Zhao et al., 2019a. Accurately weigh 0.2 g of the sample, grind it, and add Tris-HCl buffer (pH 7.5) containing 2 mmol L^-1^ EDTA, 0.3 mmol L^-1^ sucrose, 0.3 mol L^-1^ mannitol, and 0.5 g L^-1^ polyvinylpyrrolidone. One unit activity of H^+^-ATPase was defined as the release of 1 μmol of phosphorus per minute. One unit activity of COX was defined as an increase of 0.1 in absorbance per minute at 510 nm. Measure protein content using the Bradford method (Bradford, 1976).

### 2.6 Determinations of H_2_O_2_, malondialdehyde (MDA), VC, flavonoids, total phenols, reduced glutathione (GSH), and oxidized glutathione (GSSG) contents

The content of H_2_O_2_ was determined according to the method of Li et al., 2021. Weigh 0.2 g of the sample accurately, mix it with phosphate buffer (pH 7.0) and ethanol. After centrifugation, add the supernatant to the titanium salt color reagent (20% v/v sulfuric acid solution containing 5% w/v titanium sulfate). Measure the absorbance at 415 nm after 10 min of incubation. The content of MDA was determined by assay kit (item number: BC0025, Beijing Solarbio Science & Technology Co., Ltd.). Ascorbic acid was determined by the molybdenum blue colorimetric method (He et al., 2025). The standard curve established in the experiment was y = 0.0038x - 0.0109, with R² = 0.9989. Flavonoid was determined by the aluminum colorimetric method (Duan et al., 2023). The curve is y = 0.7147x - 0.0014, with R^2^ = 0.9997. Total phenols was determined by the Folin-Ciocalteu method (Casajús et al., 2021). The curve is y = 5.1042x + 0.0015, R^2^ = 0.9995. The GSH and GSSG contents were determined according to the method of Orrantia-Araujo et al., 2019. The measurements were performed using Shimadzu LC-20A. The column is XBridge^®^ Amide (5 μm, 4.6×250 mm), with the detection wavelength set at 365 nm.

### 2.7 Determinations of glutathione reductase, SOD, POD, and CAT activities

Activities of SOD, POD, CAT, and GR were determined by assay kit (item number: BC5165, BC0095, BC4785, and BC1165, Beijing Solarbio Science & Technology Co., Ltd.). Weigh 0.2 g of the sample accurately, grind it in liquid nitrogen, and then proceed according to the standard kit instructions.

### 2.8 Determinations of glucose, fructose, and sucrose contents

Homogenize 1 g of frozen fruit (from about 300 g fruit) with 50 mL of ultrapure water, ultrasonic treatment for 0.5 h, centrifuge (4°C, 4500 ×*g*) for 20 min, and collect the supernatant. Then, pass through the 0.22 micrometer filter membrane. The measurements were performed using Shimadzu LC-20A. The column is XBridge^®^ Amide (5 μm, 4.6×250 mm; XBridge Ltd.), The mobile phase is acetonitrile: water (78:22, v/v). The findings were reported as milligram of glucose, fructose, and sucrose per gram, in accordance with the method of Soyseven et al., 2022.

### 2.9 Determinations of hexokinase, phosphofructokinase, acid invertase, neutral invertase, sucrose phosphoric acid synthetase, and sucrose synthase activities

Activities of HK, PFK, AI, NI, SPS, and SS were determined by assay kit (item number: BC0740, BC0535, BC0565, BC0570, BC0600, and BC4785, Beijing Solarbio Science & Technology Co., Ltd.). Weigh 0.2 g of the sample accurately, grind it in liquid nitrogen, and then proceed according to the standard kit instructions.

### 2.10 Statistical analyses

Independent samples *t*-tests was performed to determine statistical significance at the *p*<0.05, *p*<0.01 and *p*<0.001 levels using IBM SPSS Statistics 27. The results were expressed as the mean ± standard deviation (SD).

## 3 Results

### 3.1 Effect of 1-MCP treatment on the phenotypic characteristics of winter jujube

**Figure 1:**
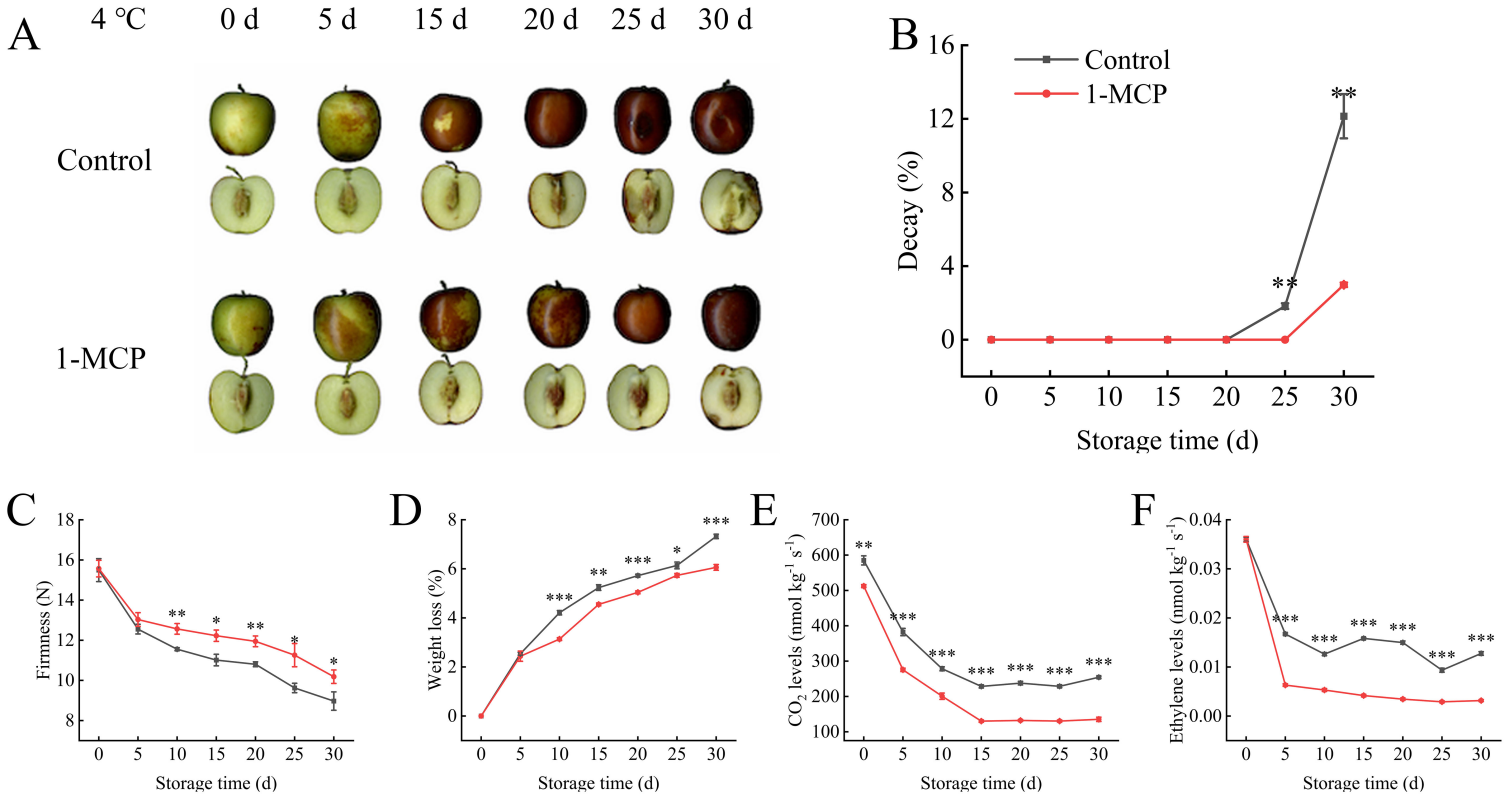
Effects of 1-methylcyclopropene (1-MCP) treatment on the phenotypic characteristics of winter jujube under 4°C storage. **(A)** Phenotype, **(B)** decay, **(C)** firmness, **(D)** weight loss, **(E)** CO_2_ levels, **(F)** ethylene levels. Statistical significance was determined by the student’s t-test: *p< 0.05, **p<0.01 and ***p<0.001.

During the first 15 days of storage, the phenotype of the winter jujube in both groups was similar, exhibiting a decrease in luster and a change color change from green to red. However, differences in phenotype became apparent at 25 days (**Figure 1A**). The fruits in the control group showed obvious atrophy, accompanied by black spots, and began to rot and deteriorate. This was further confirmed by the rate of decay (**Figure 1B**). Significant differences in firmness reduction and weight loss increase emerged at 10 days, and the 1-MCP treatment showing lower values than the control (**Figures 1C, D**). Following 1-MCP treatment, the differences in respiratory rate and ethylene production compared to the control appeared earlier than the differences in firmness and weight loss (**Figures 1E, F**). At 30 days, the respiratory rate and ethylene production in the 1-MCP treatment exhibited decreases of 46% and 82% decrease relative to day 0, whereas the control experienced a 35% and 54% reduction. The 1-MCP treatment maintained a lower and more stable respiratory rate and ethylene production throughout the storage period.

### 3.2 Effect of 1-MCP treatment on the energy metabolism of winter jujube

**Figure 2:**
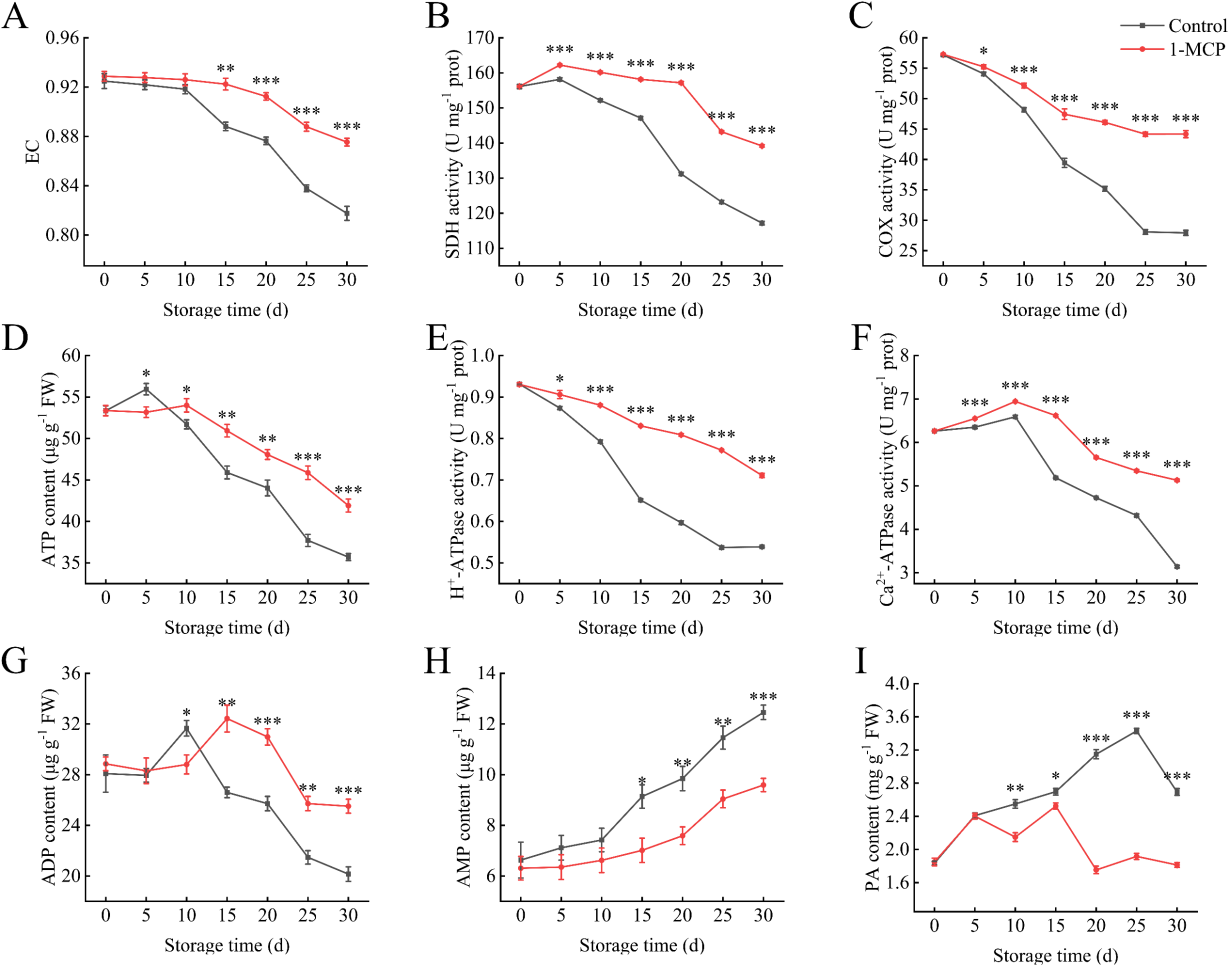
Effects of 1-MCP treatment on the energy metabolism of winter jujube under 4°C storage. **(A)** Energy charge (EC), **(B)** succinate dehydrogenase (SDH), **(C)** cytochrome c oxidase (COX), **(D)** ATP, **(E)** H^+^-ATPase, **(F)** Ca^2+^-ATPase, **(G)** ADP, **(H)** AMP, **(I)** pyruvate (PA). Statistical significance was determined by the student’s t-test: *p< 0.05, **p<0.01 and ***p<0.001.

After 1-MCP treatment, the EC was maintained to a certain extent. However, during the first 10 days of storage, there was no significant difference compared to the control (**Figure 2A**). At 30 days, the EC in the 1-MCP treatment was 7% higher than that of the control. The ATP content in the 1-MCP treatment was lower than that in the control after 5 days of storage but become significantly higher than that of the control thereafter (**Figure 2D**). A similar trend was observed in the changes in ADP content (**Figure 2G**). At 30 days, the ATP and ADP contents in the 1-MCP treatment were 17% and 27% higher, respectively, than those in the control.

The activities of SDH and COX exhibit a downward trend during the storage period. However, treatment with 1-MCP significantly preserves these enzyme activities (**Figures 2B, C**). At 30 days, enzyme activities increased by 19% and 58% compared to the control. A similar trend is observed in the changes of H+-ATPase activity (**Figure 2E**). Ca^2+^-ATPase activity slightly increased during the first 10 days of storage but then declined rapidly thereafter. Application of 1-MCP mitigated this decline. At 30 days, the activity levels in the 1-MCP treatment and control groups were 82% and 50% of the initial activity at day 0, respectively (**Figure 2F**).

The AMP content gradually increased with the extension of storage time (**Figure 2H**). The 1-MCP treatment became to be significantly lower than the control after 15 days. The PA content in the control continued to rise, whereas the 1-MCP treatment maintained a low level throughout the storage period (**Figure 2I**). At 30 days, the PA content in the 1-MCP treatment was 67% of that in the control.

### 3.3 Effect of 1-MCP treatment on the sugar metabolism of winter jujube

**Figure 3:**
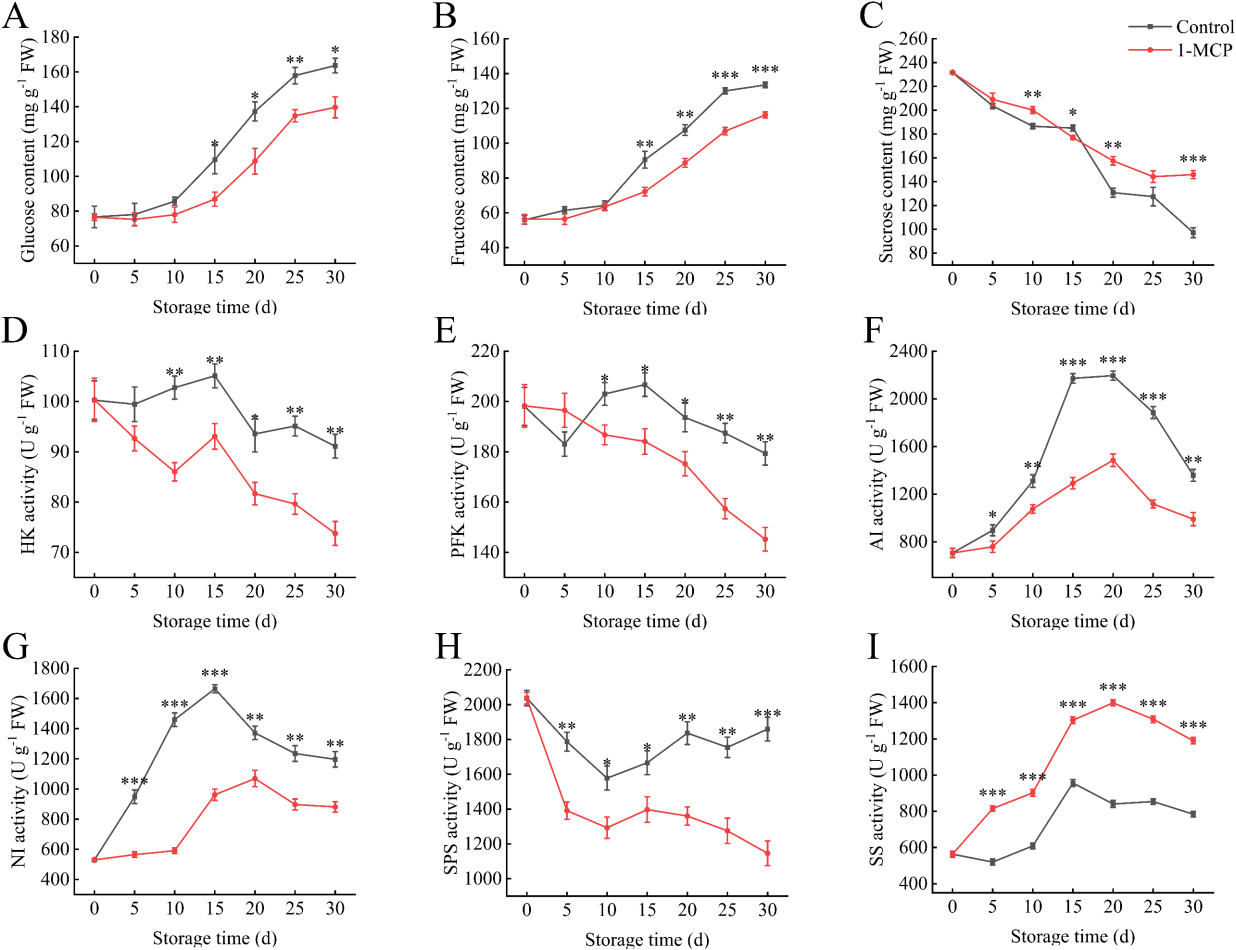
Effects of 1-MCP treatment on the sugar metabolism of winter jujube under 4°C storage. **(A)** Glucose, **(B)** fructose, **(C)** sucrose, **(D)** hexokinase (HK), **(E)** phosphofructokinase (PFK), **(F)** acid invertase (AI), **(G)** neutral invertase (NI), **(H)** sucrose phosphoric acid synthetase (SPS), **(I)** sucrose synthetase (SS). Statistical significance was determined by the student’s t-test: *p< 0.05, **p<0.01 and ***p<0.001.

The glucose and fructose contents of the winter jujube in both groups exhibited similar increasing patterns over the storage period (**Figures 3A, B**). The 1-MCP treatment showed significantly lower sugar levels compared to the control starting at 15 days. The HK activity was significantly lower than the control at 10 days (**Figure 3D**). At 30 days, the HK activity in the 1-MCP treatment was 81% of that in the control. A similar trend is also present in the changes of PFK activity (**Figure 3E**).

The sucrose content continuously decreases during storage; however, 1-MCP treatment can delay the loss (**Figure 3C**). The NI and AI activities were significantly reduced at treated with 1-MCP (**Figures 3F, G**). The NI and AI activities in control reached their peak during the 15 days to 20 days, and the sucrose content decreased the most during this period. Following 1-MCP treatment, the SPS activity decreased (**Figure 3H**), whereas the SS activity increased under the same conditions (**Figure 3I**).

### 3.4 Effect of 1-MCP treatment on the antioxidant capacity of winter jujube

**Figure 4:**
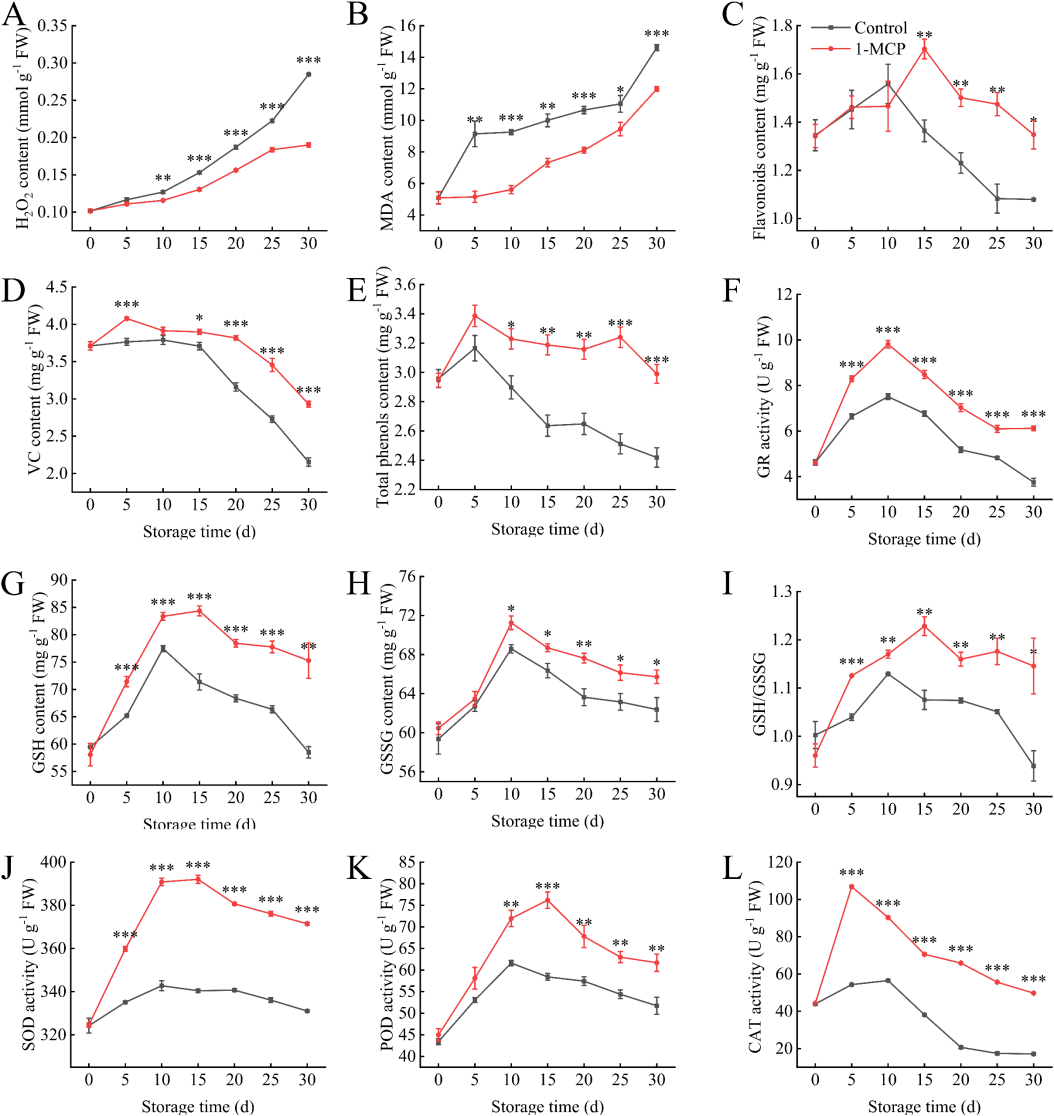
Effects of 1-MCP treatment on the antioxidant capacity of winter jujube under 4°C storage. **(A)** Hydrogen peroxide (H_2_O_2_), **(B)** malonaldehyde (MDA), **(C)** flavonoids, **(D)** vitamin C (VC), **(E)** total phenols, **(F)** glutathione reductase (GR), **(G)** reduced glutathione (GSH), **(H)** oxidized glutathione (GSSG), **(I)** GSH/GSSG ratio, **(J)** superoxide dismutase (SOD), **(K)** peroxidase (POD), **(L)** catalase (CAT). Statistical significance was determined by the student’s t-test: *p< 0.05, **p<0.01 and ***p<0.001.

The H_2_O_2_ and MDA contents were significantly lower than those of the control after10 days of 1-MCP treatment (**Figures 4A, B**). At the end of storage, the H_2_O_2_ and MDA contents were 67% and 82% of those in the control, respectively. Furthermore, 1-MCP treatment effectively maintained higher levels of VC, flavonoids, and total phenols contents throughout the storage period (**Figures 4C–E**). Following 1-MCP treatment, the activities of SOD and CAT significantly increased throughout storage (**Figures 4J, L**). The activity of POD began to improved significantly after 10 days (**Figure 4K**). Compared with the control, the 1-MCP treatment resulted in increases of 12%, 190%, and 19% in SOD, CAT, and POD activities, respectively, at 30 days.

During storage, the GR activity in the 1-MCP group was significantly higher than that in the control group (**Figure 4F**). The GR activity in both groups increased during the first 10 days and then declined. The GSH/GSSG ratio in the treated group gradually increased, peaked on 15 days, then declined and stabilized (**Figure 4I**). Throughout the storage period, the ratio in the treated group remained significantly higher than in the control group, with a 22% difference observed by 30 days.

### 3.5 Principal component analysis and correlation analysis

**Figure 5:**
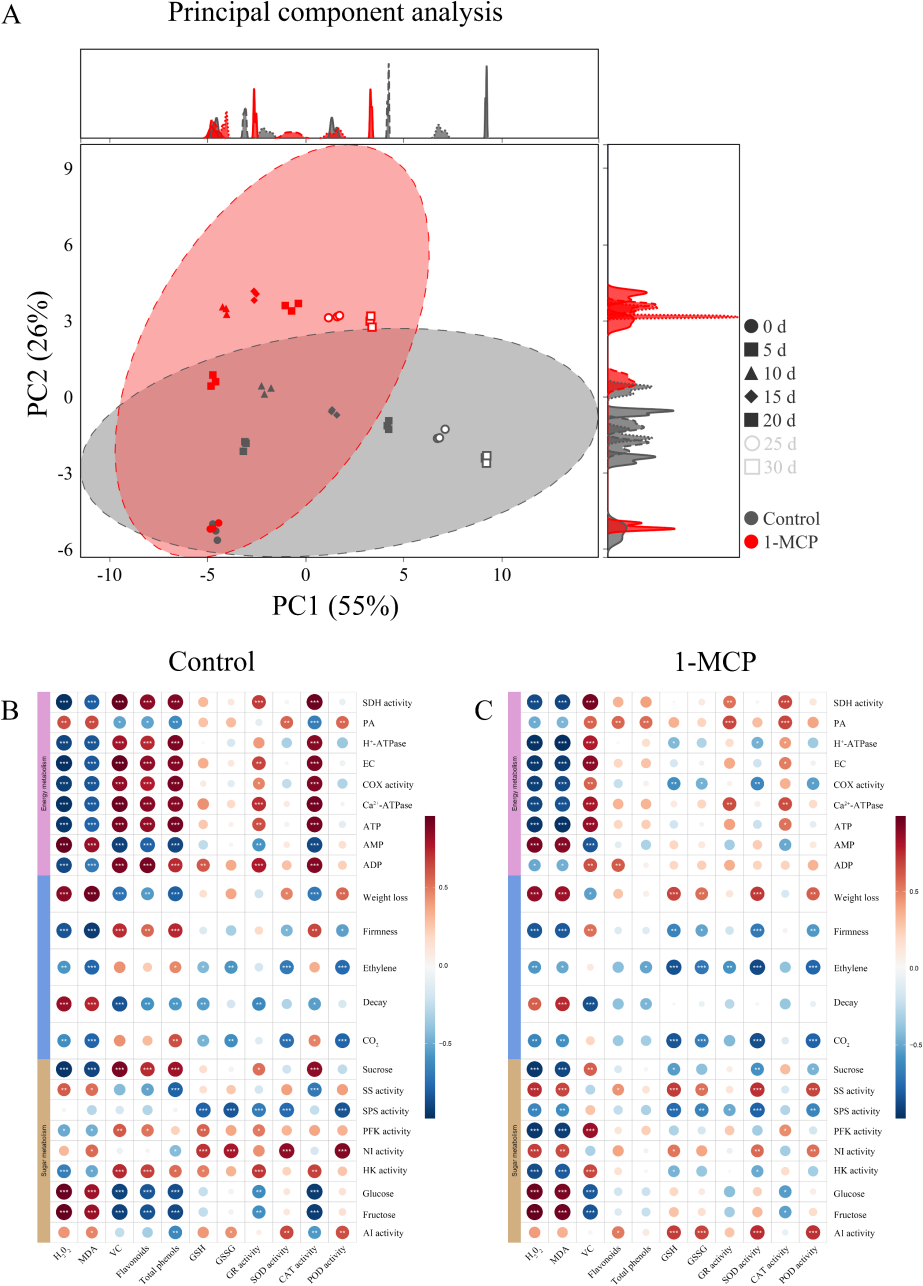
Principal component analysis (PCA) and correlation analysis of winter jujube fruit. **(A)** PCA of the control and the 1-MCP, **(B)** correlation analysis of the control, **(C)** correlation analysis of the 1-MCP. Pairwise comparisons of factors are shown in the dot, with a color gradient denoting Pearson’s correlation coefficient. *p < 0.05, **p < 0.01, ***p < 0.001.

The cumulative contribution rates of principal component 1 (PC1) and principal component 2 (PC2) are 55% and 26%, respectively (**Figure 5A**). Biological replicate samples from both the 1-MCP treatment and the control showed good repeatability at different time points. At day 0, the samples from both the 1-MCP treatment and control were located in the third quadrant. From days 15 to 30, the control sample were distributed in the fourth quadrant, while the 1-MCP treatment samples were found in the first and second quadrants. The substantial differences between these two groups strongly indicate that 1-MCP treatment has a significant effect on winter jujube.

The correlation between energy metabolism, sugar metabolism, and antioxidant capacity on winter jujube during storage was analyzed, as shown in **Figures 5B, C**. Postharvest H_2_O_2_ and MDA contents were significantly positively correlated with glucose and fructose contents in sugar metabolism and significantly negatively correlated with the activities of HK and PFK. Following 1-MCP treatment, the negative relationship between H_2+_O_2+_ and MDA contents and the activities of HK and PFK became stronger. In the control group, the contents of H_2_O_2_ and MDA were a significantly positively correlated with the AMP and PA contents, and a significantly negatively correlated with other energy metabolism indicators (ATP, ADP, COX, SDH, H^+^-ATPase, Ca^2+^-ATPase, and EC). The 1-MCP treatment weakened the positive correlation between H_2_O_2_ and MDA contents as well as ADP content, while the negative correlation with PA content shifted to a positive one. In the control group, the activities of GR and CAT were significantly negatively correlated with glucose and fructose contents in the sugar metabolism, significantly positively correlated with sucrose content and HK activity, significantly positively correlated with ATP and ADP contents, the activities of SDH, COX, Ca^2+^-ATPase, and EC in the energy metabolism, and significantly negatively correlated with AMP content. However, these correlations were weakened following 1-MCP treatment. In the control, the activities of SOD and POD were significantly positively correlated with the activities of AI and NI in the sugar metabolism, significantly negatively correlated with SPS activity, and significantly positively correlated with PA content in energy metabolism. The 1-MCP treatment enhanced the correlation between the activities of SOD and POD and AI activity, while weakening the correlations with NI activity and PA content.

## 4 Discussion

Postharvest senescence of agricultural products is a major factor determining their market value. Effectively delaying this deterioration is crucial for preserving quality, extending shelf life, and expanding market reach (Pang et al., 2024; Pei et al., 2022). Excessive ethylene production at low-temperature leads to an increase in ROS, accelerating cellular aging (Tao et al., 2019). The ethylene inhibitor 1-MCP, recognized for its safety and environmental friendliness, has attracted considerable research interest for its role in postharvest preservation (You et al., 2022). This study investigated the effects of 1-MCP treatment on postharvest energy metabolism, sugar metabolism, and antioxidant capacity of winter jujube, providing theoretical guidance and practical insights for the development of preservation technologies.

The ROS play regulatory roles in diverse physiological processes. Compared with normal temperature, low-temperature slows down metabolism, reduces ROS accumulation, and alleviates oxidative stress (Lu et al., 2025). Both enzymatic and non-enzymatic antioxidant systems provide protection against oxidative damage in plants (Tan et al., 2020). The CAT, SOD, and POD can effectively reduce the oxidative substances in cells. Additionally, non-enzymatic antioxidants such as VC, flavonoids, and total phenols further mitigate oxidative damage in fruit (Zhang et al., 2023). In this study, following the application of 1-MCP significantly reduced oxidative stress markers such as MDA and H_2_O_2_ in the fruit, while markedly enhancing the activities of antioxidant enzymes (SOD, POD, CAT, and GR), thereby alleviating the internal oxidative state. Similar effects of 1-MCP application have been observed in flat peach and purple sweet potato (Zheng et al., 2023; Nie et al., 2024). The less oxidative internal environment helped protect non-enzymatic antioxidants from degradation. Even when measured under *in vitro* higher oxidative conditions, the preserved levels of non-enzymatic antioxidants further confirmed that 1-MCP effectively mitigated oxidative stress (Rudenko et al., 2023). However, the senescence of fruit and vegetables is not only closely linked to antioxidant activity but also involves a complex network of metabolic processes (Zhang et al., 2025b).

Energy status is intricately linked to ROS metabolism. The scavenging of ROS and the repair of membrane damage are energy-demanding processes. Therefore, the depletion of major energy molecules, such as ATP and ADP, ultimately drives the rapid deterioration of postharvest quality (Wang et al., 2025b; Yang et al., 2023). This phenomenon has also been observed in studies on Agaricus bisporus and pitaya (Shang et al., 2025; Du et al., 2025). In this study, a decline in energy status, indicated by decreases in EC, ATP, and ADP contents, was observed during storage. Treatment with 1-MCP effectively mitigated this decline and was concurrently associated with a significant enhancement in the activities of key antioxidant enzymes (SOD, POD, and CAT). A similar effect has also been observed in the postharvest treatment of peppers and plums with thymol (Li et al., 2025; Tian et al., 2025). As essential components of both the TCA cycle and the ETC, SDH, and COX are located on the inner mitochondrial membrane. Therefore, their activities directly reflect the state of cellular energy metabolism (Chen et al., 2021; Wang et al., 2020). Treatment with 1-MCP enhances the activities of SDH and COX, thereby accelerating intramitochondrial electron and proton transfer and promoting ATP synthesis. This effect of 1-MCP has also been observed in strawberry fruit (Wang et al., 2025c). By catalyzing ATP hydrolysis, the H^+^-ATPase provides energy required for proton extrusion against its electrochemical, maintaining the membrane potential and regulating pH. Similarly, Ca^2+^-ATPase performs analogous functions for calcium ions, thus preventing cytotoxicity and ensuring cellular homeostasis. Moreover, cellular calcium ion (Ca^2+^) flux is intricately linked to the production of ROS (Li et al., 2023b; Mahato et al., 2023). By enhancing the activities of these enzymes, 1-MCP treatment promoted energy production and the transport of ions (H^+^ and Ca^2+^), thereby improving cellular homeostasis. This was evidenced by the reduced levels of H_2_O_2_ and MDA, indicating lower ROS and improved plasma membrane integrity.

The process of cellular respiration, which is central to energy metabolism, occurs through either aerobic or anaerobic pathways. The key difference lies in the fate of pyruvate produced in the cytosol by glycolysis. In aerobic respiration, pyruvate enters the mitochondria and is oxidized through the TCA cycle and the ETC, resulting in the production of a large amount of ATP. In contrast, during anaerobic respiration, the decomposition products of pyruvate do not enter the ETC but can only generate a small amount of ATP through substrate-level phosphorylation (Zhang et al., 2022b). After harvest, winter jujube fruit enters a state of heightened anaerobic respiration, leading to the accumulation of anaerobic metabolic products at elevated levels (Li et al., 2023a). In this experiment, the CO_2_ release and the PA content in winter jujube fruit treated with 1-MCP were significantly lower compared to the control. Concurrently, higher energy levels (ATP, ADP, and EC) and enhanced activities of SDH and COX were observed. These findings suggest that 1-MCP treatment improved aerobic respiration and the activity of related enzymes, enabling more efficient ATP production from a relatively small amount of PA, thereby sustaining normal physiological metabolism. In contrast, untreated fruit relied heavily on anaerobic respiration to meet metabolic energy demands. This reliance led to accelerated glycolysis, resulting in greater PA production for anaerobic metabolism and a corresponding rise in CO_2+_ release. Glucose is broken down through the glycolysis process into ATP and ADP, while fructose and sucrose enter the glycolysis after conversion, providing energy for physiological metabolism (Zhu et al., 2023). In this study, following 1-MCP treatment, the HK activity, responsible for phosphorylating glucose and fructose, decreased, while the activities of NI and AI enzymes involved in sucrose decomposition, were also reduced. PFK, a key irreversible regulatory enzyme in glycolysis that catalyzes the conversion of fructose-6-phosphate to fructose-1, 6-bisphosphate (Lim et al., 2019), similarly exhibited decreased activity after treatment. SPS synthesizes sucrose from fructose 1, 6-bisphosphate, although its activity decreased after treatment. In contrast, SS converts fructose into sucrose, and its activity was enhanced following treatment. These combined changes contributed to a restriction in the overall rate of glycolysis. Similar findings were reported in studies involving additional carbon dioxide treatment of strawberries and the anaerobic treatment of kiwifruit (Zhang et al., 2022b; Wang et al., 2025d).

## 5 Conclusion

**Figure 6:**
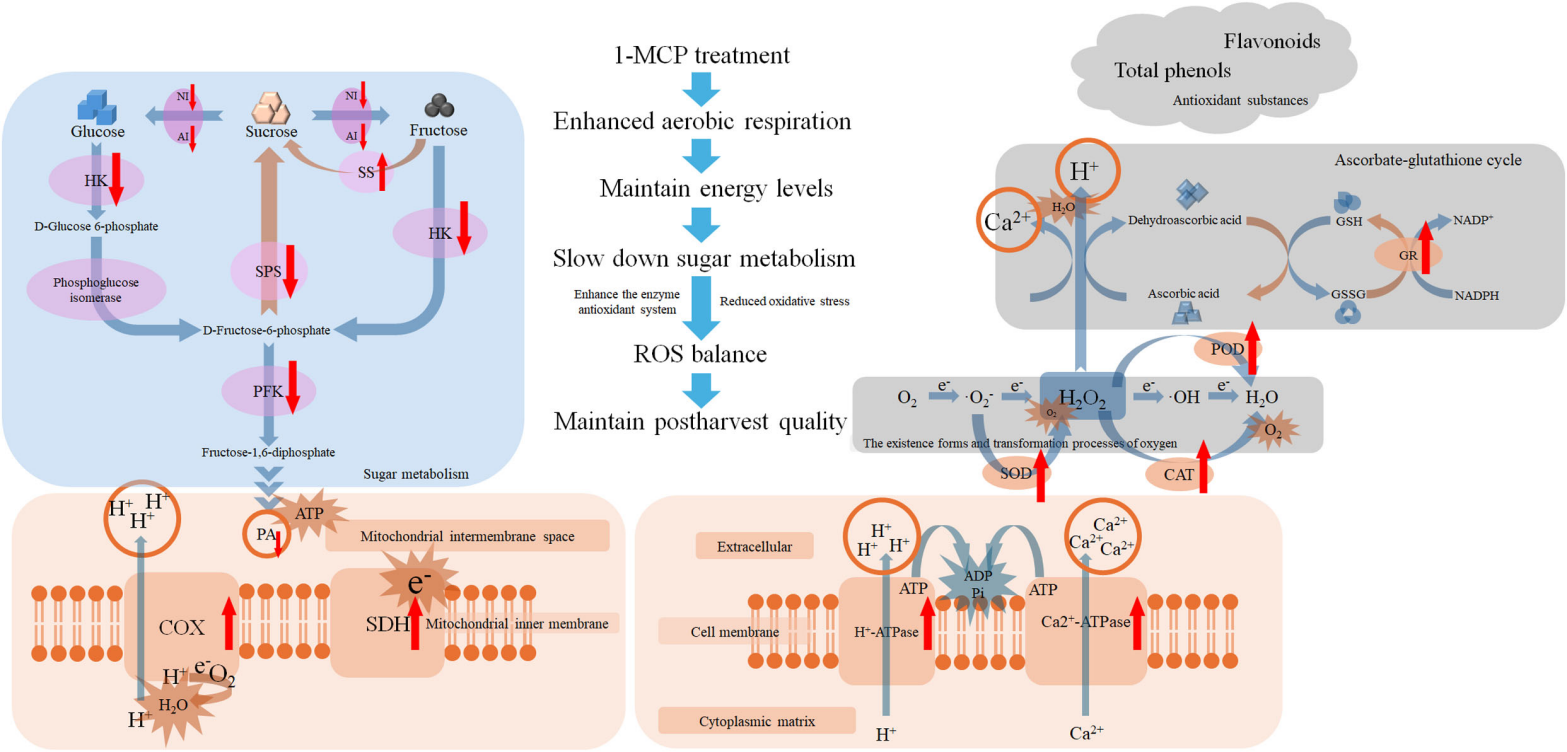
Diagram of the potential model for 1-MCP maintaining postharvest quality in winter jujube fruit. 1-MCP treatment can slow down sugar metabolism, improve energy metabolism, reduce pyruvate demand, increase energy levels, enhance ion transport capacity, and also enhance antioxidant enzymes activities as well as the ascorbate-glutathione (ASA-GSH) cycle level, in order to alleviate oxidative damage and maintain product quality. The upward red arrow denotes upregulation or increase, while the downward red arrow indicates downregulation or decrease.

Following 1-MCP treatment, energy metabolism was enhanced, and aerobic respiration was promoted, enabling the limited breakdown of PA to meet physiological demands. Consequently, reliance on anaerobic respiration and the accumulation of its associated metabolites were reduced. Furthermore, the decomposition and synthesis of glucose, fructose, and sucrose were effectively regulated, resulting in a slowdown of sugar metabolism. The activities of antioxidant enzymes were enhanced, oxidative damage has been reduced, and the levels of non-enzymatic antioxidants were well maintained. Overall, our findings demonstrate that 1-MCP treatment effectively delayed the decay and firmness loss in winter jujube, increased energy levels, slowed down sugar metabolism, enhanced antioxidant capacity, and reduce oxidative stress. **Figure 6** presents a potential working model illustrating how improves the postharvest quality of winter jujube.

## Data Availability

The original contributions presented in the study are included in the article/supplementary material. Further inquiries can be directed to the corresponding authors.
